# Mousepost 2.0, a major expansion of the resource

**DOI:** 10.1093/nar/gkad064

**Published:** 2023-02-10

**Authors:** Steven Timmermans, Jolien Vandewalle, Claude Libert

**Affiliations:** VIB Center for Inflammation Research, 9052 Ghent, Belgium; Department of Biomedical Molecular Biology, Ghent University, Ghent, Belgium; VIB Center for Inflammation Research, 9052 Ghent, Belgium; Department of Biomedical Molecular Biology, Ghent University, Ghent, Belgium; VIB Center for Inflammation Research, 9052 Ghent, Belgium; Department of Biomedical Molecular Biology, Ghent University, Ghent, Belgium

## Abstract

The Mousepost 1.0 online search tool, launched in 2017, allowed to search for variations in all protein-coding gene sequences of 36 sequenced mouse inbred strains, compared to the reference strain C57BL/6J, which could be linked to strain-specific phenotypes and modifier effects. Because recently these genome sequences have been significantly updated and sequences of 16 extra strains added by the Mouse Genomes Project, a profound update, correction and expansion of the Mousepost 1.0 database has been performed and is reported here. Moreover, we have added a new class of protein disturbing sequence polymorphisms (besides stop codon losses, stop codon gains, small insertions and deletions, and missense mutations), namely start codon mutations. The current version, Mousepost 2.0 (https://mousepost.be), therefore is a significantly updated and invaluable tool available to the community and is described here and foreseen by multiple examples.

## INTRODUCTION

The mouse is the most important and most widely used mammalian model organism species. It takes a prominent place in biomedical and genetic research, both for fundamental research and for disease treatment/drug development. It has a rich history in research, dating back more than a century, but the sequencing and publication of the mouse reference genome has marked the real start of the modern genetic and genomic sequence-based research (1). The year 2022 marks the twentieth anniversary of the publication of the first draft of the mouse reference genome, derived from the C57BL/6J strain (2).

Mouse research is usually performed using inbred mice, which are, by definition, homozygous over their entire diploid genome and belong to a certain inbred strain. Several hundreds of such inbred strains have been described, the most popular ones being commercially available at several sources. Some of these inbred strains exhibit strain-specific phenotypes, such as resistance to lipopolysaccharides in the mice belonging to the C3H/HeJ strain (3). Also, many examples have been described, illustrating that mutations introduced in mice via embryonic stem cells or CRISPR/Cas9 exhibit very significantly different phenotypes, depending on the mouse inbred strain in which the mutation was introduced (4). Such data suggest the existence of strain-specific genome variations that should be mapped to the nucleotide level, to understand the role, function and mechanisms of these elements in the aforementioned observations (5,6).

During the last two decades, significant advancements were made in the quality of the mouse C57BL/6J reference genome sequence (7) and projects towards obtaining sequence data from other inbred lines were also started. The Mouse Genomes Project (MGP), initiated and maintained by the Wellcome Sanger Institute, is the primary effort towards whole genome shotgun sequencing and variant detection in mouse inbred lines (8,9). The initial release of the MGP data in 2011 contained 36 popular and frequently used inbred strains (selected in collaboration with the mouse research community) (9). These data were available as complete genome assemblies for a subset of the strains, and ‘variant call format’ (VCF) files containing information of single-nucleotide polymorphisms (SNPs) as well as small insertions and deletions (indels) for all strains.

Our research group has used the nucleotide level data released by the MGP to build the ‘Mousepost’ resource (an acronym of mouse polymorphic sequence tags; https://mousepost.be). Initially started from a project to provide an overview of the mutations in the wild-derived inbred strain SPRET/EiJ (10), this endeavour was expanded to all protein-coding transcripts in all MGP sequenced strains and we released the first version of the data and tool in 2017 (11). This web-based tool provided strain-specific protein sequence information for the 36 strains in the MGP. We processed the data from the VCF files and only retained variants causing non-synonymous amino acid substitutions on a per codon basis. The first release of Mousepost (Mousepost 1.0) included a classification of the protein sequences [stop-gain (SG), stop-loss (SL) or others] and data export and search functions, making it an excellent complementary resource of the MGP (7). We expanded Mousepost several times with novel functionalities, such as variants in the reference strain compared to the others and direct pairwise comparisons between strains (12,13). Furthermore, we also made a derivate of Mousepost, Ratpost, providing the same type of information for rat inbred strains (14,15).

Based on the popular use of Mousepost 1.0 and recent significant changes in (i) the underlying sequence data in the reference genome, (ii) the MGP sequences and (iii) the addition of new sequenced strains, the construction of a new version as Mousepost 2.0 was mandatory. This new Mousepost 2.0 version adds data from 16 new strains, bringing the total number of strains present to 52. The refinements to the MGP data cause the addition of many new events and removal of others compared to the previous version. In this paper, we disclose and discuss the main additions and removals to the Mousepost database and describe changes to the processing pipeline. Moreover, we have now also added a new class of sequence polymorphisms, which cause start codon (SC) mutations, as a new class and have also processed genome sequence patches on the reference genome meaning that we involved the incremental sequence changes of the last 5 years that were not part of the previous version. The multitude of changes in the Mousepost 1.0 database are illustrated by several examples that illustrate the power of the new Mousepost 2.0 release, available at https://mousepost.be, and the tool in general.

## MATERIALS AND METHODS

*Variant data* were obtained from the ftp site of the MGP (8) as a VCF file containing SNPs and indels from all included strains, as release REL-2005. We used the Picard tools (https://broadinstitute.github.io/Picard/) ‘LiftoverVcf’ command with the appropriate chain file from the University of California, Santa Cruz (UCSC) to reprocess the mm10 annotated VCF file to the new mm39 coordinates and structural annotation. This VCF was processed to remove heterozygous and low-quality calls and split per strain. All files were processed into a MySQL relational database, which holds the DNA sequence for every variant position in all strains, including C57BL/6J.

Structural reference annotation. We used the GRCm39 version of the mouse genome reference strain (C57BL/6J), which we obtained from the Genome Reference Consortium (GRC) ftp site. The structural annotation used was the complete annotation, version M28 from the gencode resource (16), as in ‘gene transfer format’ (GTF) format.

*Mousepost data processing* was done with the Mousepost pipeline as described in (12). The only difference to the pipeline itself was that the conserved domain database for the rps-blast was updated to the release of 22 February 2021.

*PROVEAN* (Protein Variation Effect Analyzer) (17) score calculation was performed on a compute cluster using saved supporting gene sets whenever possible and the same blast database. This allows us to keep −2.5 as a score threshold below which the mutation is predicted to be deleterious to protein function with a balanced accuracy of 80% as described by the PROVEAN authors.

Gene Ontology. Information for mouse was downloaded from the Gene Ontology (GO) Consortium website (www.geneontology.org/). We processed this file to update the GO terms for all genes in our dataset.

*Sequence alignments* were made with the needle algorithm from the EMBOSS tools (18) in case of pairwise alignments or with the MUSCLE aligner (19) for multiple sequence alignments. Multiple sequence alignments were used to obtain majority vote consensus sequences to compare the reference C57BL/6J genome too.

## RESULTS AND DISCUSSION

### The Mousepost 2.0 pipeline data

**Figure 1.**
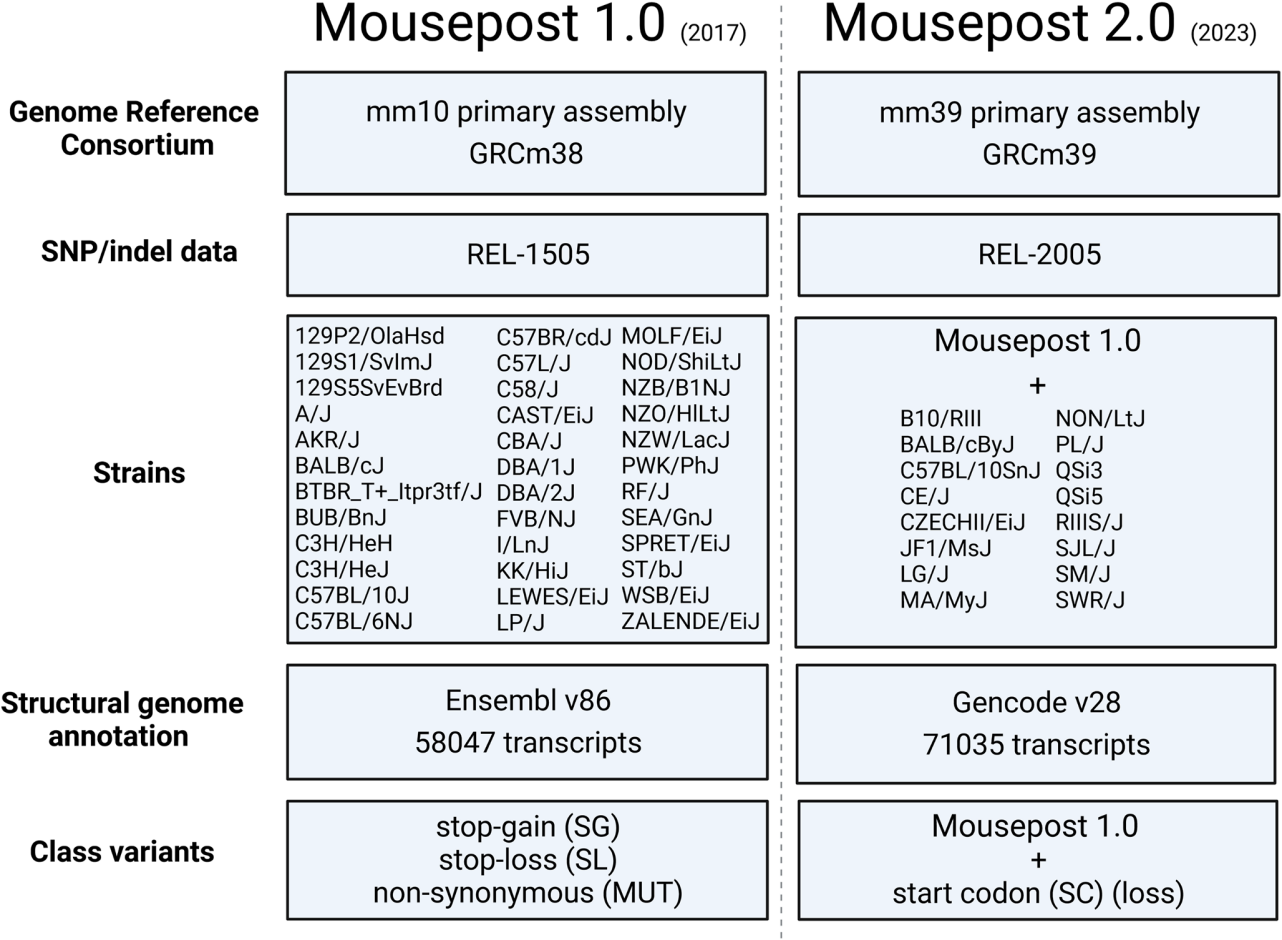
Schematic representation of the differences between Mousepost 1.0 and Mousepost 2.0, the updated version described in this work.

All source datasets used to create the Mousepost 2.0 resource were updated: the SNP/indel data, the C57BL/6J reference genome sequences and the reference genome annotation. A complete comparison of the data and workflow used in the original Mousepost and Mousepost 2.0 can be found in Figure 1. Briefly, the following updates were performed: (i) In order to obtain the most relevant dataset, the genome annotation was changed to the most recent release of the mouse genome, which is mm39 at the time of writing. As the variants were called using the mm10 genome annotation, we used Picard tools (https://broadinstitute.github.io/Picard/), specifically the command LiftoverVcf, with the ‘mm10tomm39’ chain file obtained from the UCSC ftp servers to lift the mm10 VCF file to the mm39 mouse genome assembly (20). We then used the gencode v28 release (21) as the structural annotation from which transcripts were obtained, changed from Ensemble release 85. (ii) The SNP/indel data were updated from REL-1505 to REL-2005, which is the most recent release available on the ftp site (4). This REL-2005 release contained data of a total of 52 mouse inbred strains, which is 16 more than the REL-1505 version that was used in the initial Mousepost version (7). The newly added strains are B10/RIII, BALB/cByJ, C57BL/10SnJ, CE/J, CZECHII/EiJ, JF1/MsJ, LG/J, MA/MyJ, NON/LtJ, PL/J, QSi3, QSi5, RIIIS/J, SJL/J, SM/J and SWR/J.

### Processing and annotation of transcripts

The variant VCF file was first converted to the mm39 version of the mouse reference genome. This was done using the LiftoverVcf command from the Picard tools software and the mm10 to mm39 UCSC chain file obtained from https://hgdownload.soe.ucsc.edu/goldenPath/mm10/liftOver/. The transcript GTF files and lifted VCF file were converted to ‘browser extensible data’ file format with BEDTools (22), in order to process the files with BEDOPS (23). To minimize processing time, only relevant transcripts and events were selected: (i) we made the intersect of the variants with the transcripts to retain only those variants that overlapped a transcript location; and (ii) to select relevant transcripts, the opposite overlap was made, with only transcripts overlapping one or more variants being kept. Furthermore, all heterozygous events, where one of the alleles matches the reference (C57BL/6J), low-quality events (filtered on the FI tag, with FI = 1 events retained) and non-protein-coding transcripts or pseudogene transcripts were filtered out of the dataset. This provided a total of 71 035 transcripts across all strains to be processed by the Mousepost 2.0 pipeline, as described in (11,13), an increase of 12 988 compared to the original Mousepost release. Briefly, we assembled the untranslated sequences (5′UTR and 3′UTR) and coding sequence (CDS) from the structural annotation and sequence data. We also included the sequence data from the so-called reference mouse (C57BL/6J) ‘genome patches’. These are 87 sequence scaffolds with sequence data that would lead to coordinate changes if integrated in the mm10 reference genome. We mapped the coordinates to the standard chromosomes and used the patch sequence as a replacement where applicable. The sequences from the patches were given precedence in the construction of the reference version of the UTR, CDS and eventual translated sequences. Strain-specific sequences were translated and compared for classification. The number of classes was expanded from the three original ones [SG, SL and non-synonymous variants (MUT)] with a fourth specific class, namely SC variants. Indeed, the initial release of Mousepost did not include these variants fully appropriately in any of the other three classes, and although only a small group, such SC losses can be very deleterious and thus have a high impact on protein function. Novel MUT class variants were also subjected to a prediction of their potential effect on protein function with the PROVEAN tool (17). All PROVEAN predictions were made using the same database used in the initial version of Mousepost. As always, a PROVEAN score of −2.5 or lower suggests a significant loss of function of the protein involving a sequence variation.

### Updated statistics

First, the REL-2005 SNP/indel data from the MGP (from the mouse strains that are compared to the reference genome) contain significantly more data than the previously used REL-1505 (45 164 532 versus 38 700 845 in REL-1505). This leads to (i) a strong increase in number of variant transcripts compared to the reference genome. But in addition, (ii) several previously defined events are no longer present in the dataset. The main reason for such event losses is the inclusion of more accurate sequencing data, which improves SNP/indel calling and thus removes events that were previously false positive calls.

Second, use of the new version of the C57BL/6J reference genome also leads to removal of some events, but this is an almost negligible amount compared to the total number of events removed for the previous reason. On the other hand, one strain, closely related to the C57BL/6J reference strain, namely C57BL/6NJ, in the new version of the Mousepost database has no longer SL variant transcripts, while it had 46 of them in the initial version.

**Figure 2.**
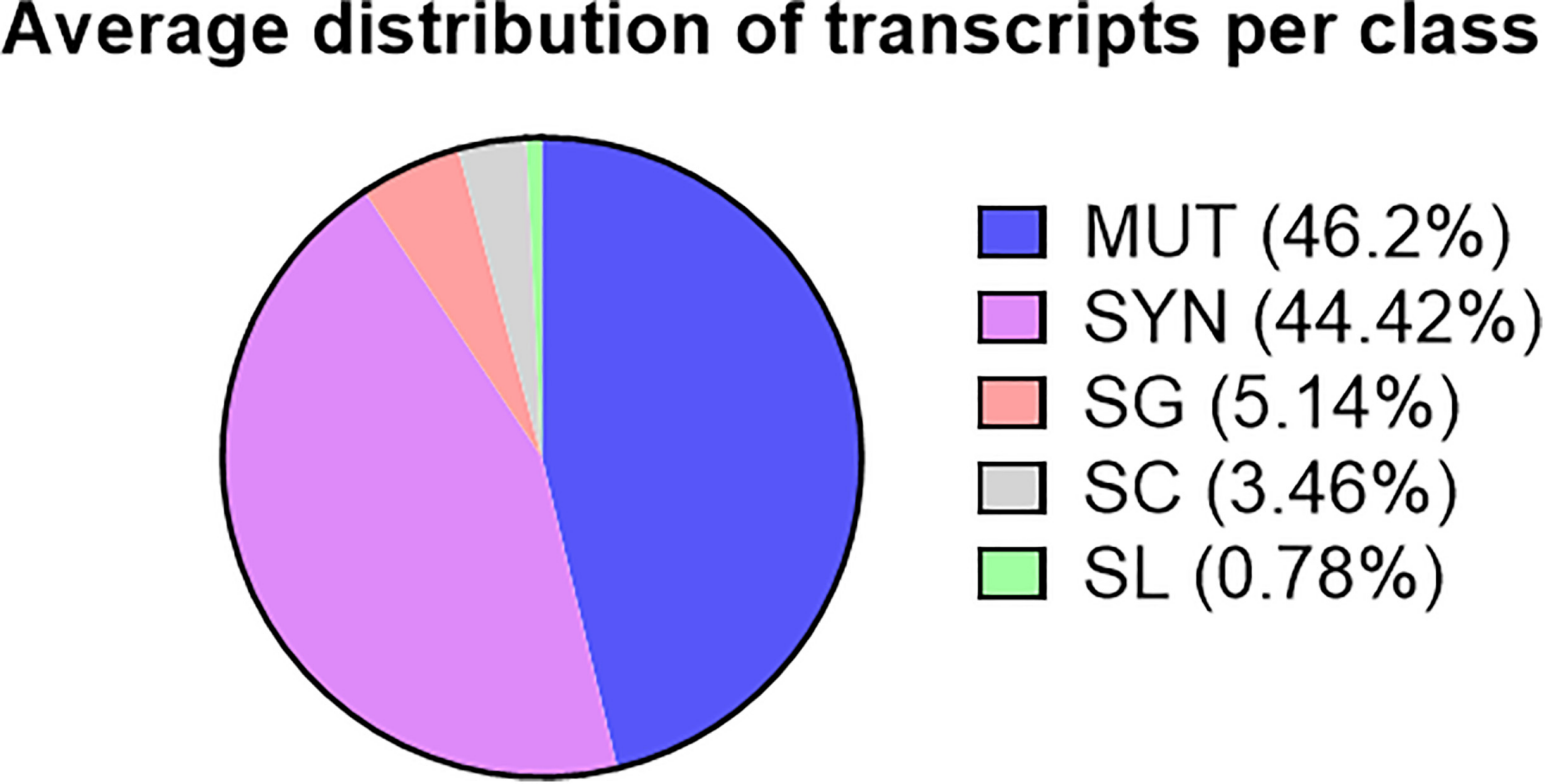
Distribution of types of variations found in transcripts across all classes. This is the total distribution of transcripts across all included strains (and compared to C57BL/6J). MUT, non-synonymous mutation; SYN, synonymous mutation; SG, stop gain; SC, start codon variant; SL, stop loss.

Third, the proportion of different classes differs markedly between both Mousepost versions, even when the new SC class is removed from the comparison (Table 1). However, the class proportions are largely the same when compared between the different strains, with strong deviations only seen in strains that are closely related to C57BL/6J and have relatively few mutated transcripts. Taking into consideration all 52 included strains (Figure 2), the largest group consists of the MUT variants, which substitute single amino acids or constitute small indels (46.20%). The second largest group of transcripts contains only synonymous events (44.42%). These are either fully silent nucleotide changes (e.g. most wobble position variants) or double mutations that cancel each other at the codon level (e.g. codon TCC→AGC, both encoding serine). Stop codon gains (nonsense variants) make up 5.14% of all variant transcripts. Furthermore 3.46% of the transcripts have an SC variation and only 0.78% belong to the SL class (Tables 2 and 3). If the synonymous class is not considered and only transcripts with mutations leading to amino acid changes are taken into account, then 1.40% of those have an SL mutation, 6.23% have a mutation in the canonical SC, 9.24% have gained a premature stop codon (SG) and 83.13% have a non-synonymous mutation that is in no way related to start or stop codons.

**Table 1.** Comparison between the number of transcripts per class in the new and old versions of Mousepost

	Mousepost 1.0			Mousepost 2.0		
**Strain**	SG	SL	MUT	SG	SL	MUT
129P2/OlaHsd	179	125	6176	351	52	9144
129S1/SvImJ	173	127	6151	321	52	9028
129S5SvEvBrd	159	116	6052	324	46	8934
A/J	185	100	6100	364	40	8845
AKR/J	181	112	6021	335	29	9039
BALB/cJ	158	108	5651	321	41	8388
BTBR_T+_Itpr3tf/J	200	108	6262	298	48	7478
BUB/BnJ	163	116	6551	330	43	9548
C3H/HeH	144	104	5247	324	35	9081
C3H/HeJ	188	126	6218	343	41	9225
C57BL/10J	21	54	556	40	6	758
C57BL/6NJ	12	47	84	20	0	144
C57BR/cdJ	96	76	2954	190	18	4408
C57L/J	85	71	2842	175	16	4135
C58/J	99	90	3781	203	32	5356
CAST/EiJ	515	258	16 812	709	164	23 568
CBA/J	149	103	4565	352	44	9503
DBA/1J	180	119	6351	354	39	9258
DBA/2J	194	119	6428	362	39	9450
FVB/NJ	183	114	6228	263	31	9217
I/LnJ	206	122	6675	406	48	9621
KK/HiJ	192	125	6327	347	53	9615
LEWES/EiJ	230	139	9123	447	64	13 282
LP/J	204	131	6328	364	48	9402
MOLF/EiJ	528	266	15 884	850	163	22 495
NOD/ShiLtJ	184	115	6364	347	40	9602
NZB/B1NJ	162	112	6170	317	49	9676
NZO/HlLtJ	176	112	6310	306	46	9340
NZW/LacJ	193	121	6821	197	40	9949
PWK/PhJ	583	278	16 566	648	166	23 377
RF/J	174	110	6471	360	52	9556
SEA/GnJ	177	115	6195	361	43	9163
SPRET/EiJ	1055	459	21 891	1264	359	30 657
ST/bJ	178	115	7703	356	51	9085
WSB/EiJ	257	144	9246	293	57	13 494
ZALENDE/EiJ	291	156	10 481	364	61	15 139

This table compares the 36 mouse inbred strain present in both Mousepost versions.

**Table 2.** Number of polymorphic events on protein-coding transcripts, per class, for the 16 new mouse inbred strains, as compared to C57BL/6J

**Strain**	SC	SG	SL	MUT
B10.RIII	75	69	13	1560
BALB/cByJ	304	320	41	8413
C57BL/10SnJ	55	45	6	742
CE/J	340	274	48	10 609
CZECHII/EiJ	862	700	178	23 844
JF1/MsJ	803	662	182	23 399
LG/J	285	342	42	9379
MA/MyJ	307	342	36	8743
NON/LtJ	307	321	49	8951
PL/J	327	334	50	9331
QSi3	342	367	50	9399
QSi5	347	332	42	9237
RIIIS/J	323	358	49	9420
SJL/J	352	241	47	9532
SM/J	327	378	60	9695
SWR/J	342	344	48	9534

**Table 3.** SC variants in the 36 strains in the original Mousepost 1.0 release

Strain	Start codon	Strain	Start codon
129P2/OlaHsd	295	DBA/2J	353
129S1/SvImJ	310	FVB/NJ	33
129S5SvEvBrd	288	I/LnJ	325
A/J	305	KK/HiJ	328
AKR/J	627	LEWES/EiJ	383
BALB/cJ	310	LP/J	341
BTBR_T+_Itpr3tf/J	250	MOLF/EiJ	713
BUB/BnJ	323	NOD/ShiLtJ	348
C3H/HeH	304	NZB/B1NJ	307
C3H/HeJ	328	NZO/HlLtJ	314
C57BL/10J	63	NZW/LacJ	318
C57BL/6NJ	28	PWK/PhJ	823
C57BR/cdJ	152	RF/J	304
C57L/J	147	SEA/GnJ	305
C58/J	207	SPRET/EiJ	1327
CAST/EiJ	813	ST/bJ	319
CBA/J	339	WSB/EiJ	461
DBA/1J	341	ZALENDE/EiJ	436

Next to the correction and expansion of the data for the 36 previously studied strains, the REL-2005 also has SNP and indel data for 16 extra strains not involved in the previous release (Figure 1). These new strains were processed in the pipeline and added to the database (Table 2), expanding the number of mouse strains to 52 that are compared to C57BL/6J. Of the added strains, two are closely related to C57BL/6J and have only a few mutated transcripts, while two other strains (CZECHII/EiJ and JF1/MsJ) are (evolutionary) far removed from C57BL/6J and are in the same order as other ‘wild-derived strains’ (e.g. SPRET/EiJ).

The addition of these strains also resulted in a complete rebuild of the ‘C57BL/6J’ section of Mousepost 2.0. In this section, we provide an overview of those transcripts that can be considered as mutated in the reference strain, because they differ in C57BL/6J compared to synthetic reference transcripts that were constructed from all strain-specific transcripts by a *per position* majority vote mechanism. For Mousepost 2.0, the synthetic reference transcripts were rebuilt completely using the REL-2005 SNP/indel data from the MGP, as well as the data from the new strains, and any newly added or removed variant information in the already included strains. The impact of this change is large: (i) There are no longer C57BL/6J-specific SG transcripts as C57BL/6NJ does not have any SL variants compared to the reference (Table 1) and, overall, the number of SG C57BL/6J variants drops to 27 (from 38). (ii) The number of SL variants increases from 121 to 277 and the number of MUT class transcripts with a PROVEAN score of ≤−2.5 is 2684 in Mousepost 2.0 versus 1892 in Mousepost 1.0. All transcripts where the protein sequence was different in C57BL/6J from the synthetic consensus reference were included as missense transcripts in the database.

### Mousepost use and applications

In this section, we will provide direct examples of Mousepost use at https://mousepost.be. We will highlight the changes to the previous version by the new data and provide a step-by-step overview as to how the tool can be used to obtain the data of interest.

#### A general overview of the number of transcripts per class and per strain

On the welcome page of the tool, a short description is provided as well as four submenus. The ‘data overview’ item gives an overview of how many distinct transcripts and genes can be found in each of the variant classes of all included strains, compared to the reference C57BL/6J. This table can be updated with the application of filters for SG, SL and missense (MUT) classes. For SG and SL, this filter is the maximal and the minimal length ratio, respectively, of the strain-specific transcript compared to the reference (C57BL/6J) one. For MUT class genes, this is the maximal value of the PROVEAN score below which at least one scored variant must fall; i.e. if there are three variants in the transcript, and the filter is set to ‘−5’, at least one of those must have a score of −5 or lower to be included in the overview. The numbers that are shown in the table also act as internal links: for each strain, the numbers of genes and transcripts displayed are direct links to the strain-specific lists of variants immediately filtered to return the actual transcripts that are behind the number shown in the main table. Compared to the previous version of Mousepost, the main change is the inclusion of a new class, SC variants.

The other two submenus (‘Compare to Reference’ and ‘Deviations in C57BL/6J’) are a set of guided links to the main functions, all of which can be accessed from the top menu bar. The submenus will guide the user to the correct page for performing the desired query, through a set of descriptions. The main functions, which will be discussed in detail, are as follows: (i) *per strain lists* (‘Lists’) of each variant type, optionally filtered by chromosomal region, which is also what the number of transcripts/genes from table in the previous paragraph links to; (ii) *gene, gene location and gene function (ontology) searches* (‘Search’), to search for information for specific genes, genomic regions or gene sets related to specific functions in *multiple strains* at the same time; and (iii) the ability to perform *direct pairwise comparisons* of any two strains (‘Pairwise’). Next to these comparisons, there is also the option (iv) to obtain gene lists *per mutation type* of those genes that are considered to be *mutated in the reference genome*, obtained by comparison with a synthetic reference (‘C57BL/6J’).

#### Obtaining a list of strain-specific transcripts

##### The new SC variant class

**Figure 3.**
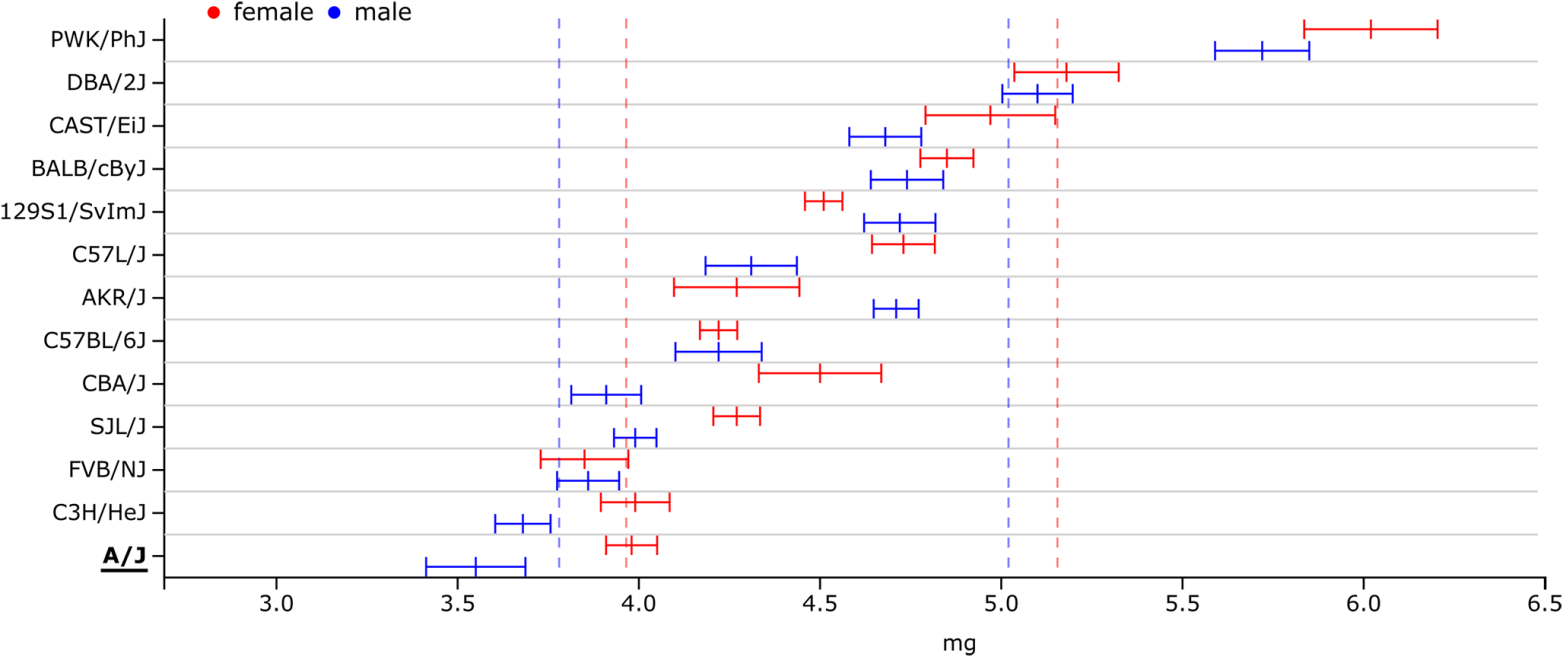
Distribution of ventricle weight (median with complete measured range) (left and right) of the mouse heart in those mouse strains in which this has been measured, corrected for the total body weight of the mice. The A/J strain has the overall lowest relative ventricle weight, with especially male A/J mice being on the extreme low end of the distribution. Image adapted from the mouse phenome database (https://phenome.jax.org/).

One of the novelties of Mousepost 2.0 is the addition of the SC variant class. All strains have several SC variants assigned to them. We assume that these will mostly result in significant loss of function of the protein that should normally be produced, which is why we have chosen to include this set as a separate class. We can use gene set enrichment analysis to find phenotypes and pathways that are related to these genes. Due to the low number of such genes, no statistically significant enrichments could be found; however, there was a trend to lipid metabolism-related functions and cardiac development and/or function. The *A/J* strain has both these trends and, according to the mouse phenome database, also displays phenotypical characteristics in accordance with the predicted functions: a relatively low level of free fatty acids circulating in the blood compared to other tested inbred strains (Supplementary Figure S1) and a very low heart size compared to body weight (the lowest measured) (24) (Figure 3). In order to perform such analyses, the list of SC transcripts for the A/J strain can be easily obtained from the Mousepost tool. Here, we can use the ‘Lists’ function of the menu bar and fill in the strain of interest (SOI), A/J, the variant type of interest and the SC variants. This gives a complete list of all SC variants in A/J and can be easily exported for further processing, such as gene set enrichment analyses. This list may also be obtained through the main overview table on the tool homepage. As previously described, the numbers of transcripts per strain and per class act as internal hyperlinks, which lead to the strain-specific transcript page. As the data are retrieved from the database with HTTP_GET requests, direct passing of the search/filter parameters is possible. Thus, the links directly give the results for the correct strain with application of the selected filters for length ratio or PROVEAN score; other filters such as lost protein domains or location restrictions must be set on the ‘Lists’ page.

When studying the SC mutants for the A/J stain, we find a potential link to cardiac development in these mice to the *Mybpc3* gene (25,26). This gene has an SC loss that affects all transcripts (the methionine codon is mutated to an isoleucine codon, M1I). The protein will likely be truncated from the N-terminal side, as there is a second, in-frame, SC just 8 AAs downstream of the normal one, which would result in the partial removal of an ‘immunoglobulin-like domain’ from the N-terminal part of the protein (normally from AA position 2→103). The question remains how severely this would affect protein function and whether the downstream ATG can be used as a substitute SC.

##### Stop codon and non-synonymous variants

**Figure 4.**
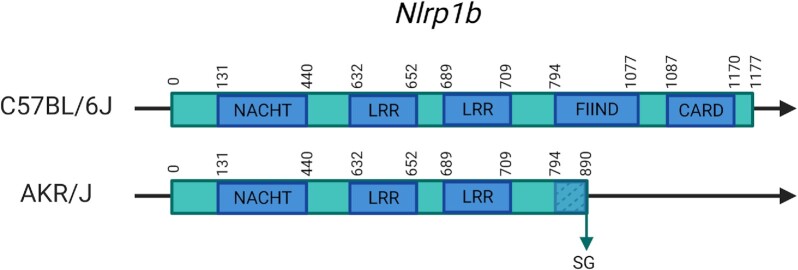
Representation of the new SG variant in Nlrp1b in AKR/J mice and its effects on the protein, which is in C57BL/6J 1177 AAs in size. Domains are added based on UniProt/InterPro. FIIND and CARD domains are, respectively, partially or completely affected by the truncation. NACHT, nucleoside triphosphatase (NTPase) domain; LRR, leucine-rich repeat; FIIND, function to find domain; CARD, caspase activation and recruitment domain.

Obtaining lists of transcripts for the other classes works for any SOI in almost the same manner. For stop codon variants, the strain and length ratio (>1 for SL and <1 for SG) must be selected. In the case of SG, it is also possible to check a ‘domains’ option, which will also give an overview of which domains are part of the truncated region in the result set. The results are returned as a table with, for each transcript, the length in the C57BL/6J and the SOI version, and a visual ratio as a red/green bar where the red part is the part that is truncated (SG) or added (SL) to the reference sequence. This update increases the number of variants in each class and caused the reclassification of several genes and transcripts. The latter is mostly due to inclusion of new SG mutations, and an example is seen when retrieving a listing for the AKR/J SG variants since one of the new genes recovered in this release is *Nlrp1b*. This gene is related to the sensitivity to anthrax lethal toxin, to which the AKR/J strain has previously been shown to be resistant (27). This resistance has been linked to *Nlrp1b* (28), the gene coding for a part of the Nlrp1 inflammasome. *Nlrp1b* loss of function in other strains also confers this resistance to the toxin (27–29). This gene has multiple mutations, several of which were included in the previous Mousepost database and have PROVEAN scores (−7 and −9) predicting loss of function. However, multiple new events in this gene were included from REL-2005, one of which causes an SG mutation in all coding transcripts of this gene. This SG results in the loss of 287 AAs (24%) truncating the protein from 1174/1177 to 887/890 AAs depending on the transcript. This will very likely result in a partial loss of the ‘function to find’ (FIIND) domain and total loss of the ‘caspase activation and recruitment domain’ (CARD) domain (Figure 4 and Supplementary Figure S2). There is one caveat the user must take into account when querying SG or SL variants. In case of SG variants, depending on where the nonsense mutation has occurred, there is a (high) possibility of ‘nonsense-mediated decay’ (NMD) (30). *In vivo* or *in vitro*, when a nonsense mutation occurs ‘early’ in a transcript, the mRNA may be recognized as aberrant and be degraded immediately and so no meaningful amount of truncated peptide will remain. As it is not possible to determine which proteins may (not) undergo NMD, we have chosen to report the length of the encoded protein that is affected by the SG variants. These reported data do not depend on NMD, but users working with mice should be careful when working *in vivo* or *in vitro* with SG variants as the mRNAs can be degraded and the truncated protein absent. In addition, a mechanism (called ‘non-stop decay’) exists to degrade SL mRNA (31,32), but is mostly applicable to those transcripts lacking any stop codon at all, which does not occur that often.

Non-synonymous variants can be queried per strain by selecting ‘MUT’ and specifying a maximal PROVEAN score. The result table obtained here lists all transcripts that have at least one variant with PROVEAN score lower than the selected cut-off. Next to the gene and transcript, the total number of variants in the protein sequence is displayed as well as the score of the lowest scoring variant.

In all cases, the result table contains several links to more information: the Ensemble IDs are internal links to a ‘details’ page providing in detail information for the transcript and strain and the final column contains external links to UniProt, Ensembl, Mouse Genome Informatics (MGI) and UCSC Genome Browser.

#### Searching the database

Next to per strain and per variant type listings, it is also possible to perform an extensive search function consisting of three main options: a gene of interest (GOI), with optional filters, a region of interest and a GO term. In all cases, results are returned as a table, with results grouped by variant type and displaying the strain, transcript ratio and length ratio, for SG, SL and SC, or lowest PROVEAN score, for MUT transcripts. For the GOI search, one or more gene names can be entered, and the official gene name or an Ensembl gene ID can be used. The main application is to study whether one or more GOI(s) have variants (of any type) in any strain (by default all, but any can be selected). As an illustration, we will search for a previously described mutation in the *Tlr3* gene (25) in one of the strains that was added in this update: the *Tlr3* P369L variant found in CZECHII/EiJ. Using the search function with the query ‘Tlr3’ as input, and filtering on strains and mutation types, we are able to easily recover and confirm the *Tlr3* mutation, a P369L change (33), which ruins the function of TLR3, similar to the deleterious *Tlr4* P712H change in the genome of strain C3H/HeJ (3).

CZECHII/EiJ mice are resistant to diet-induced obesity since they remain lean when they are fed a high-fat diet (HFD) for 8 weeks [mouse phenome database, Paigen1 dataset (34); Supplementary Figure S3]. Searching the Mousepost 2.0 dataset using the GO search function from the website as well as data from the literature for genes related to diet-induced obesity resulted in transcripts from two genes being affected. One is the *Akap1* gene, which has 5 AA substitutions, and is known in relation to this phenotype (35). However, none of the AA changes individually have a PROVEAN score of −2.5 (lowest is −1.4) or lower, and are thus not predicted to be deleterious, but all of them combined may still impact function. The use of the GO search function using the GO term ‘regulation of appetite’ yields a large result table, which was narrowed down by entering CZECHII in the table search/filter field. One of the genes found here, as well as in the literature, is the cholecystokinin-coding gene (*Cck*). Mice that have a loss of function of this gene were shown to be resistant to HFD-induced obesity (36). In CZECH/EiJ mice, *Cck* shows an SG mutation leading to the production of a truncated peptide of the canonical transcript. The resulting deletion is small, only 10 AAs preceded by a 4 AA frameshift of a normally 115 AA protein, but this affects the active peptide region, which indeed is known to involve the very C-terminal end of the protein. The final option is the possibility to search a genomic region. This is an extension on the location filter that can be optionally enabled when obtaining strain-specific gene lists. The main difference between the location filter in the strain-specific lists and the location search is that the search is performed across all strains and variant types and without any cut-offs, e.g. for the PROVEAN score. When this functionality is used with a certain region of interest, such as can be obtained from quantitative trait loci (QTL) studies, it is possible to obtain candidate genes for the trait investigated that also have at least one variant in the strain used. In the case of CZECHII/EiJ, an *in silico* QTL study from 2012 for nurturing ability (37), in which the authors showed a very low ‘average daily weight gain’ of pups for this strain, nine genes were identified as candidates in the QTL analysis. Based on Mousepost 2.0, we can associate two of these (*Fn1* and *Rbm44*) with protein-coding variants in the CZECHII/EiJ inbred line when using the regions provided to search our tool database and comparing the results.

#### The updated reference and C57BL/6J variants

The change from mm10 to mm39 version of the reference genome has directly influenced the variants included in the database: several previously included variants have been removed as they had become irrelevant, indicating that these were in fact false positive calls. Furthermore, a large number of new variants were included. The largest impact of these facts can be found in the number of variants per strain being higher in this release as reported previously and in the ‘C57BL/6J’ part of Mousepost. In this part of the tool, we present those transcripts, divided per class, that were found to be deviant in the reference genome (C57BL/6J) compared to the synthetic consensus reference. To determine this, we compared this genome against a synthetic reference as described previously (12). When selecting one of the four mutation classes, a table of C57BL/6J variants is returned (in the same form as for the strain-specific lists) with one additional column: the *agreement score*. This score consists of two elements and shows how many other strains have the same variant as C57BL/6J or have a different variant at the same position. A gene that was removed in the update due to corrections to the reference genome is *Nadk2*, which was previously annotated as an SG variant exclusively in C57BL/6J. However, after Mousepost 1.0 was made available, the GRC published sequence patches, changing the reference sequence in these regions. In the new version of the mouse reference genome, mm39, the sequence in the *Nadk2* region differs from the mm10 version that was used in Mousepost 1.0. Hence, the *Nadk2* variants were present only due to (sequencing) errors and have been removed in Mousepost 2.0.

#### Pairwise comparisons

The final functionality related to protein-coding variants in the Mousepost 2.0 tool is the *pairwise comparison* function (‘Pairwise’). Here, the user can directly compare two strains, without involvement of any reference sequence. Both strains to be compared need to be selected as ‘strain A’ and ‘strain B’. To speed up the query or if one has a specific region of interest, such as from a QTL study, a genomic location filter can be added. The result table returned provides an overview of each transcript and how many AA differences there are between the selected strains. By clicking on the numbers provided in that table, the user can directly access more information: selecting the number of differences will add a window with a complete overview of non-identical positions in the form of ‘position StrainA_sequence StrainB_sequence’. The table also shows how many strains in the entire dataset have the sequence of strain A and how many have the sequence of strain B. It is important to note that these may be ‘0’ as the selected strains are not themselves counted. Clicking on these numbers will give a listing of these strains in alphabetical order, and here the chosen SOI will be included, in contrast to the number shown in the table.

#### Detailed strain-specific and gene-specific data

Each of the result tables from comparisons to the reference strain, be it from the listings per strain or search page, provides an overview summary only and contains several useful links, with the link in the final column leading to gene and genomic location information from UCSC, Ensembl and MGI. The gene names and gene/transcript IDs are internal links that lead to the ‘gene details’ page. On the ‘gene details’ page, all transcripts of the gene are listed as expandable sections giving a complete overview of all information in the database on the transcript. For SG, SL and SC classed transcripts, the information included complete AA sequence of the protein in the reference strain and selected SOI. SC transcripts also have a list of all annotated protein domains that were affected by the truncation. In case of MUT transcripts, there is a table of all variants and their PROVEAN scores.

#### A complementary resource to the MGP

The Mousepost 2.0 tool also serves as an interesting complementary tool to the data directly provided by the MGP. The MGP data can be accessed through their own variant querying site. This resource provides an extensive search function to query the database based on gene name/ID and genomic coordinates. The main difference between the MGP variant site and Mousepost 2.0 is that the MGP variant site provides a nucleotide focused result set, i.e. an overview of all variants at the level of the genome with links to the variant database from the MGI, where more information can be obtained, such as the effects on the protein, if any. Mousepost 2.0, in contrast, directly allows queries and provides information at the protein level. Mousepost 2.0 also integrates the effects of all variants, because, on several occasions, there are multiple variants affecting a single codon. This is a major difference with the information available in the MGP directly and provides significant added value. The effect is the largest for variants that are annotated as ‘stop gain’ (or nonsense) in the VCF file, as these often have a neighbouring variant (always co-occurring) resulting in a missense variant instead of a nonsense variant. A practical example is the *Cracdl* gene (ENSMUSG00000026090; reverse strand) in the A/J strain, which is annotated as having an SG variant (MGP VCF file) at genomic position chr1:37664116–37664117, with the actual mutation resulting in a GAA (E) to TAA (STOP). However, this gene also has a variant at the neighbouring position in the same strain (at chr1:37664115–37664116). By itself this variant is annotated as a missense (GAA to GTA: E→V), but both variations are always present together; thus, neither of the annotations in the VCF gives the correct real situation in A/J, which is in fact GAA→TTA resulting in E→L at the AA level, which is the single variant that Mousepost 2.0 will show, from the integration of all nucleotide variants on a *per codon* basis. MGP reports on a *per nucleotide* basis instead. We also directly incorporate exon skipping or intron retention results into the sequences for transcripts where splice sites are (predicted to) be affected. This allows us to provide comprehensive protein comparisons between the strains. However, non-coding sequence-related variants (e.g. upstream of genes) cannot be found in Mousepost 2.0, as these are only present at the nucleotide level, but they are already comprehensively annotated in the MGP. Finally, we also integrate data from all available strains into a synthetic reference to interrogate the C57BL/6J strain for mutations, which is not possible with the MGP tools. In contrast, the MGP offers complete reference genome, with all variants included, for several popular strains.

#### A complementary resource to the ‘wildmouse’ dataset

Mousepost 2.0 may also be used as a resource for users who are more interested in wild mice and wild-derived strains. It serves as a complementary resource to the ‘wildmouse’ dataset, which was published in 2016 by Harr *et al.* (38) and includes information about genomic variation in mice living in the wild, specifically individuals from the species *Mus musculus domesticus*, *Mus musculus helgolandicus*, *Mus musculus musculus* and *Mus spretus*. These data are available through their own ftp site or in the USCS Genome Browser as a public session (https://genome.ucsc.edu/cgi-bin/hgPublicSessions; search ‘wildmouse’). Using the genomic location of the variants in Mousepost, it is possible to link back to the data in the wildmouse project. The occurrence of variants from inbred strains can thus be checked in wild mice, which can be used to predict loss-of-function variants in wild mouse strains.

## CONCLUSION

This work presents a major update of the underlying data on the Mousepost 1.0 resource, based on significantly updated sequencing data of the reference strain as well as the 36 previously studied inbred strains and addition of 16 new mouse inbred strains. The expansion significantly increases the usefulness of Mousepost to the community.

## DATA AVAILABILITY

All data in this study are available from public repositories, which are listed and linked in the manuscript. All processed data are made available on the mousepost.be website.

## Supplementary Material

gkad064_Supplemental_File
