# Supplementary material for: Mousepost 2.0, a major expansion of the resource
Source: Nucleic Acids Res. 2023 Feb 10;51(4):1652–61. doi: 10.1093/nar/gkad064 (PMC9976886; doi:10.1093/nar/gkad064)
Supplement: gkad064_Supplemental_File [file gkad064_supplemental_file.pdf]

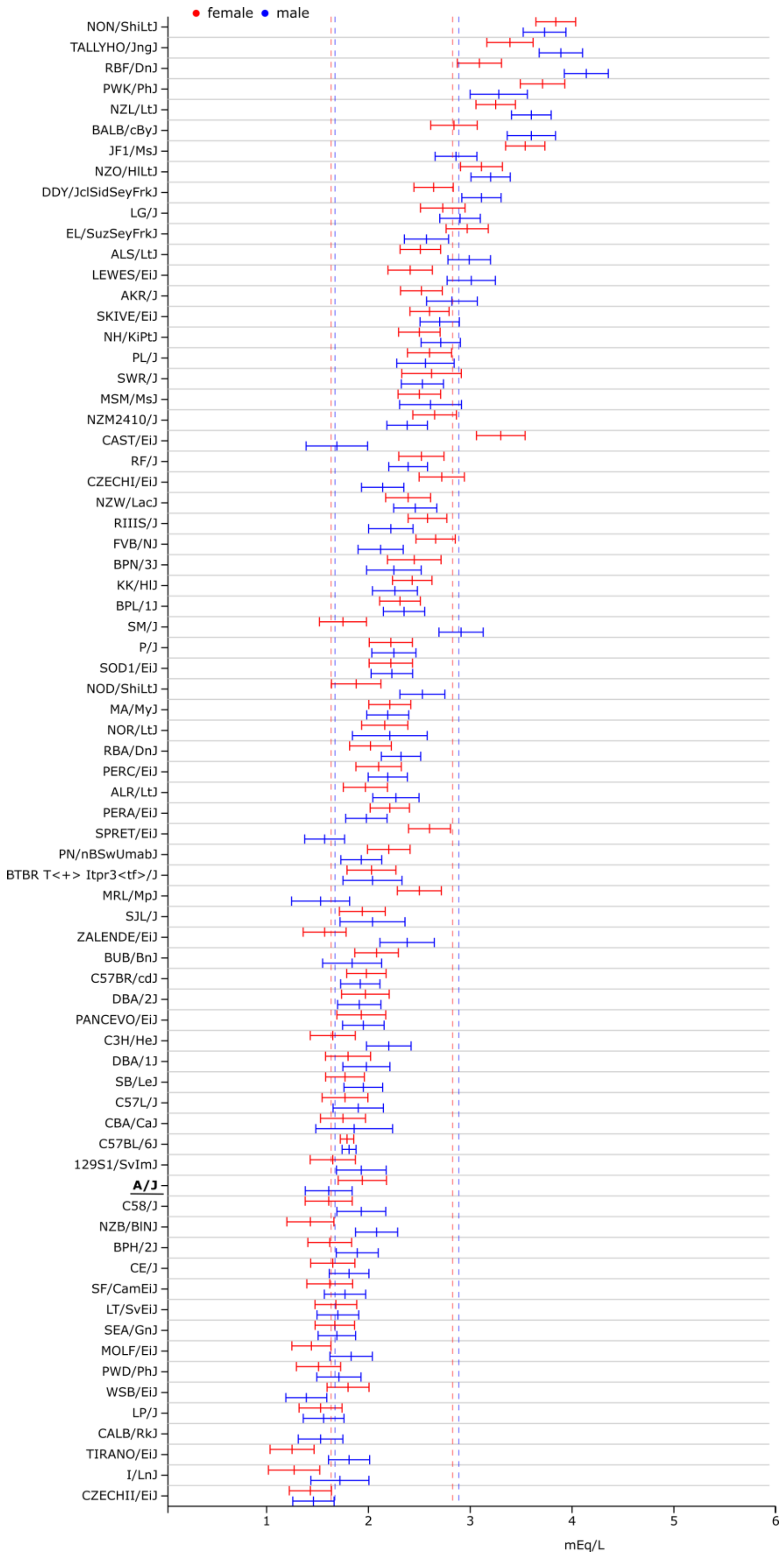

Figure S1. Circulating lipid concentration in the blood of inbred mouse strains, adapted from the mouse phenome database (https://phenome.jax.org/).

```
WT                 1 MEESQYKQEHNKKVAQDEGQEDKDTIFETIEAIEAKLMELKTNPESTFNY     50
                     ||||||||||||||||||||||||||||||||||||||||||||||||||
AKR_J              1 MEESQYKQEHNKKVAQDEGQEDKDTIFETIEAIEAKLMELKTNPESTFNY     50

WT                51 GIFPEVYMNQGEEILYPAWSLKEENLFQTFKSLRLFQKLCPRGSGNLVKK    100
                     ||||||||.|.|||||||.|||||||.|.|.||.|.||||||||.|||:|
AKR_J             51 GIFPEVYMKQREEILYPAQSLKEENLIQNFTSLPLLQKLCPRGSENLVRK    100

WT               101 SWYPCVPEEGGHIINIQDLFGPNIGTQKEPQLVIIEGAAGIGKSTLARQV    150
                     ||||||||||||||||||||||||||||||||||||||||||||||||||
AKR_J            101 SWYPCVPEEGGHIINIQDLFGPNIGTQKEPQLVIIEGAAGIGKSTLARQV    150

WT               151 KRAWMEGELYRDHFQHVFFFSCRELAQCKKLSLAELITQGQDVPTAPINQ    200
                     ||||.||:|||||||||||||||||||||||||||||:|||:||||||||
AKR_J            151 KRAWKEGQLYRDHFQHVFFFSCRELAQCKKLSLAELISQGQEVPTAPINQ    200

WT               201 ILSHPEKLLFILDGIDEPAWVLADQNPELCLYWSQTQPVHTLLGSLLGKS    250
                     |||||||||||||||||||||||||||||||:|||.||||||||||||||
AKR_J            201 ILSHPEKLLFILDGIDEPAWVLADQNPELCLHWSQRQPVHTLLGSLLGKS    250

WT               251 ILPEASFLLTTRTTALQKFIPSLPQSCQVEVLGFSDFEQEIYIYKYFAKQ    300
                     |||||.||||||||||||||||||..||||||||||..|:|.|.|||||.|
AKR_J            251 ILPEAFFLLTTRTTALQKFIPSLPMPCQVEVLGFSGIERENYFYKYFANQ    300

WT               301 IFGIKALMMVESNPVLLTLCEVPWVCWLVCNCLKKQMEQGGDVSLTSQTT    350
                     ...|.|.|||||||||||||||||||||||.|||||||||.|||||||||
AKR_J            301 RHAITAFMMVESNPVLLTLCEVPWVCWLVCICLKKQMEQGRDVSLTSQTT    350

WT               351 TAICLKYISLTIPVHHMRTQLRALCSLAAEGIWKRRTLFSESDLCKQGLD    400
                     ||||||||||||||||||||::||||||||||||||||||||||||||||
AKR_J            351 TAICLKYISLTIPVHHMRTQVKALCSLAAEGIWKRRTLFSESDLCKQGLD    400

WT               401 EDAVAIFLKTGVLQKQASSLSYSFAHLCLQEFFASMSCILEDSEERHGDM    450
                     |||||.||||||||||||||||||||||||||||::||||||||||||||
AKR_J            401 EDAVATFLKTGVLQKQASSLSYSFAHLCLQEFFAAISCILEDSEERHGDM    450

WT               451 EMDRIVETLVERYGRQNLFEAPTVRFLFGLLSKEGLKEMEKLFSCSLPGK    500
                     ||||||||||||||||||||||||||||||||||||||||||||||||||
AKR_J            451 EMDRIVETLVERYGRQNLFEAPTVRFLFGLLSKEGLKEMEKLFSCSLPGK    500

WT               501 TKLKLLWHILGKSQPHQPPCLGLLHCLYENQDMKLLTHVMHDLQGTIVPD    550
                     ||||||||||||||||||.||||||||||||||||||||||||||||||.
AKR_J            501 TKLKLLWHILGKSQPHQPSCLGLLHCLYENQDMKLLTHVMHDLQGTIVPG    550

WT               551 TDDITHTVLQTNVKHLVVRTDMELMVVTFCIQFCSHMRSLQLNMEGQQGY    600
                     .|||.|||||||||.|||:|||||||.||||||..|:|:||||||.||||
AKR_J            551 PDDIAHTVLQTNVKQLVVQTDMELMVATFCIQFYCHVRTLQLNMEKQQGY    600

WT               601 ALTAPRMVLYRWTPITNASWKILFYNLKFNSNLEGLDLSGNPLSYSAVQY    650
                     |||:||||||||||||||||:||||||||..||||||||||.||||.||.
AKR_J            601 ALTSPRMVLYRWTPITNASWEILFYNLKFTRNLEGLDLSGNSLSYSVVQS    650

WT               651 LCDAMIYPGCQLKTLWLVECGLTPTYCSLLASVLSACSSLRELDLQLNDL    700
                     ||..:.|||||||||||||||||.||||||||||||||||.||.||||||
AKR_J            651 LCKTLRYPGCQLKTLWLVECGLTSTYCSLLASVLSACSSLIELYLQLNDL    700

WT               701 CDDGVRMLCEGLRNRACNLRILRLDLYSLSAQVITELRTLEENNLKLHIS    750
                     .||||||||||||||||||.||||||.|||||||||||||||.|.|||||
AKR_J            701 GDDGVRMLCEGLRNRACNLSILRLDLSSLSAQVITELRTLEEKNPKLHIS    750

WT               751 SIWMPQMMVPTENMDEEDILTSFKQQRQQSGANPMEILGTEEDFWGPIGP    800
                     |||||.|||||||||||.|||||||||||||..||||||||||||||.||
AKR_J            751 SIWMPHMMVPTENMDEEAILTSFKQQRQQSGDKPMEILGTEEDFWGPTGP    800

WT               801 VATEVVYRERNLYRVQLPMAGSYHCPSTRLHFVVTRAVTIEIEFCAWSQF    850
                     ||||:|.|.|||||||||||||||||||.|||||||||||||||||||||
AKR_J            801 VATELVDRVRNLYRVQLPMAGSYHCPSTGLHFVVTRAVTIEIEFCAWSQF    850

WT               851 LDKTPLQQSHMVVGPLFDIKAEQGAVTAVYLPHFVSLKDTKASTFDFKVA    900
                     ||||||||||||||||||||||||||||||||||||||||
AKR_J            851 LDKTPLQQSHMVVGPLFDIKAEQGAVTAVYLPHFVSLKDT----------    890

WT               901 HFQEHGMVLETPDRVKPGYTVLKNPSFSPMGVVLRIIPAARHFIPITSIT    950

AKR_J            891 --------------------------------------------------    890

WT               951 LIYYRVNQEEVTLHLYLVPNDCTIQKAIDDEEMKFQFVRINKPPPVDNLF   1000

AKR_J            891 --------------------------------------------------    890

WT              1001 IGSRYIVSGSENLEITPKELELCYRSSKEFQLFSEIYVGNMGSEIKLQIK   1050

AKR_J            891 --------------------------------------------------    890

WT              1051 NKKHMKLIWEALLKPGDLRPALPRIAQALKDAPSLLHFMDQHREQLVARV   1100

AKR_J            891 --------------------------------------------------    890

WT              1101 TSVDPLLDKLHGLVLNEESYEAVRAENTNQDKMRKLFNLSRSWSRACKDL   1150

AKR_J            891 --------------------------------------------------    890

WT              1151 FYQALKETHPHLVMDLLEKSGGVSLGS   1177

AKR_J            891 ---------------------------    890
```

<u>Figure S2</u>. Pairwise alignment of WT reference (C57BL/6J) sequence and AKR/J sequence of the Nlrp1b protein. This alignment illustrates the many single AA differences in the sequence as well as the truncation by the new SG variant at position 881.

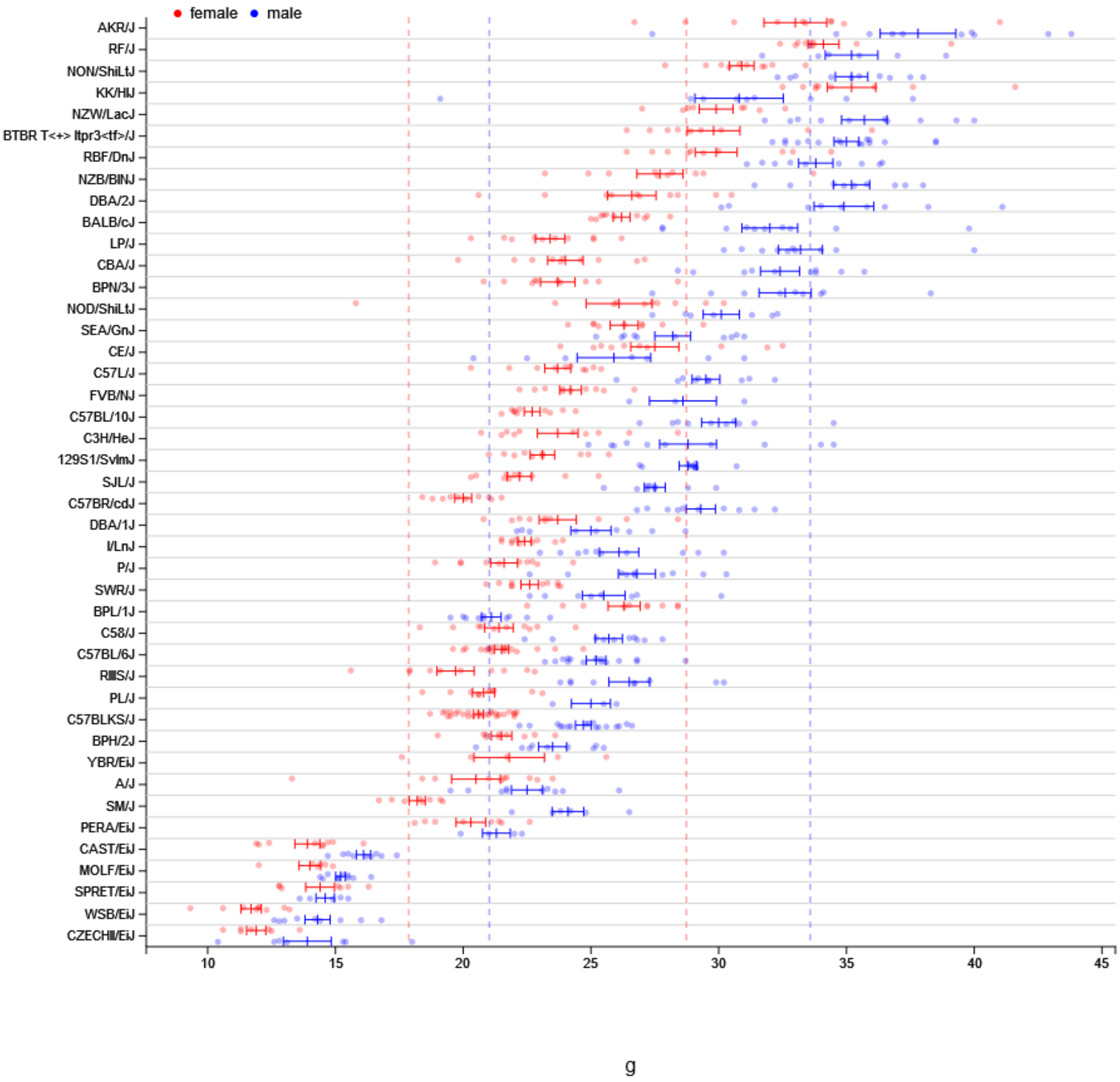

Figure S3. Body weight of inbred mouse strain after 8 weeks on a high fat diet , adapted from the mouse phenome database (https://phenome.jax.org/).
